# Clinical application of cardiac computed tomography in cardiomyopathy

**DOI:** 10.1007/s10554-025-03571-y

**Published:** 2025-11-28

**Authors:** Riccardo Cau, Marco Gatti, Jasjit S. Suri, Tommaso D’Angelo, Luca Saba

**Affiliations:** 1 Department of Radiology, Azienda Ospedaliero Universitaria (A.O.U.), di Cagliari – Polo di Monserrato s.s. 554 Monserrato (Cagliari), Cagliari, 09045 Italy; 2 University of Cagliari, Cagliari, Italy; 3 Department of Radiology, University of Turin, Turin, 10126 Italy; 4 Department of ECE, Idaho State University, Pocatello, ID 83209 USA; 5 Department of CE, Graphics Era Deemed to be University, Dehradun, 248002 India; 6 University Center for Research & Development, Chandigarh University, Mohali, India; 7 Symbiosis Institute of TechnologyInternational (Deemed University), Nagpur Campus, SymbiosisPune, India; 8 Stroke Diagnostic and Monitoring Division, AtheroPoint™, Roseville, CA 95661 USA; 9 Diagnostic and Interventional Radiology Unit, BIOMORF Department, University Hospital “Policlinico G. Martino”, Via Consolare Valeria 1, Messina, 98100 Italy

**Keywords:** Cardiomyopathy, Computed tomography, CT

## Abstract

Cardiomyopathies are a heterogeneous group of myocardial disorders characterized by mechanical and/or electrical dysfunction, typically occurring without significant coronary artery disease, hypertension, valvular, or congenital heart disease. According to current ESC guidelines, cardiovascular magnetic resonance (CMR) is a class I recommendation for the initial evaluation of patients with suspected cardiomyopathy, given its ability to provide comprehensive morphological and tissue characterization. CMR, however, may be precluded in patients owing to device incompatibility, claustrophobia, or arrhythmia- and motion-related image degradation. In this context, cardiac computed tomography (CCT), traditionally used for assessing coronary artery disease, has emerged as a valuable alternative. Technological advancements have enabled CCT to deliver not only detailed anatomical information but also functional and tissue-specific insights. The recent consensus document from the European Association of Cardiovascular Imaging underscore the expanding clinical applications of CCT, highlighting its potential role in the diagnostic work-up and phenotypic classification of cardiomyopathies. In this review, we summarize the current evidence supporting the use of CCT in patients with suspected cardiomyopathy, outline its clinical strengths and limitations, and discuss emerging developments such as artificial intelligence and radiomics, which could further enhance its diagnostic and prognostic value.

**Graphical Abstract:**

**Figure:**
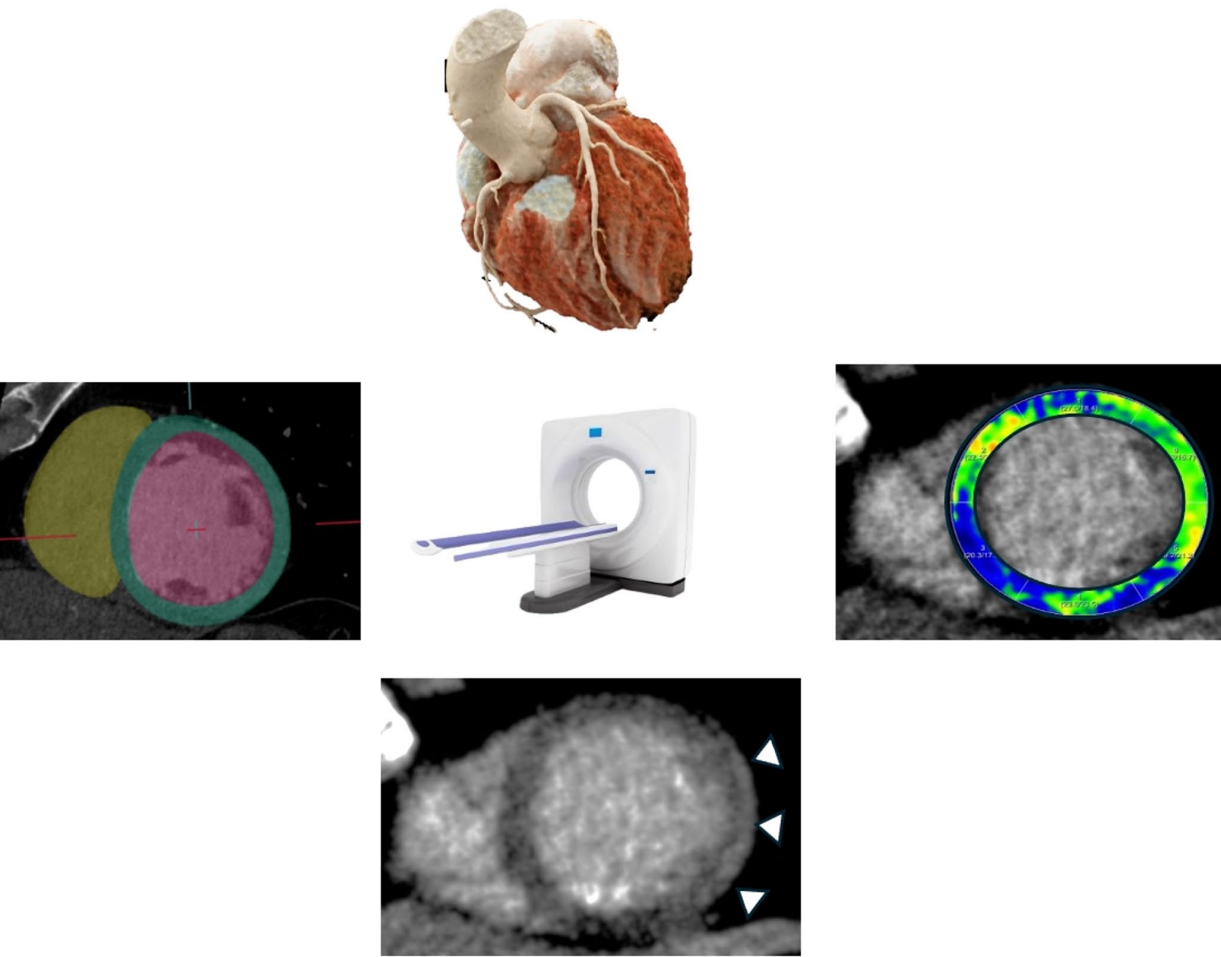
Cardiac computed tomographyis an emerging alternative to CMR, offering comprehensive anatomical, functional, and tissue characterization in suspectedcardiomyopathies

## Introduction

Cardiomyopathies encompass a diverse spectrum of myocardial diseases characterized by structural and/or functional impairment of the myocardium, often occurring without other conditions sufficient to explain the observed abnormalities [1]. These disorders represent a major cause of heart failure, arrhythmias, and sudden cardiac death, with clinical management depending significantly on accurate diagnosis and phenotypic characterization [1]. According to the current European Society of Cardiology (ESC) guidelines for the management of cardiomyopathies, non-invasive imaging plays a central role in evaluating patients with suspected cardiomyopathy, allowing the integration of anatomical, functional, and tissue-specific information into the diagnostic process. Cardiovascular magnetic resonance (CMR) remains the gold standard for myocardial tissue characterization and is recommended as a first-line imaging modality [1]. However, its applicability can be limited by contraindications such as MRI-incompatible implantable devices, claustrophobia, or limited availability but also by technical factors—persistent ventricular or supraventricular arrhythmias, rapid atrial flutter, inability to maintain adequate breath-holding, or patient motion/instability—that markedly degrade CMR image quality [2–4]. In this evolving landscape, cardiac computed tomography (CCT), traditionally used for coronary artery assessment [5–8], has emerged as a valuable adjunct or alternative imaging tool. Beyond coronary evaluation, CCT provides detailed morphological and functional information, as well as insights into myocardial tissue characteristics, thereby contributing to the phenotypic classification of cardiomyopathies [9–18]. The recent consensus document from the European Association of Cardiovascular Imaging underscores the expanding role of CCT in this setting, particularly in patients who cannot undergo CMR [19]. This review aims to summarize the clinical applications of CCT in cardiomyopathy, outline optimized imaging protocols, discuss its diagnostic contributions alongside other modalities, and explore emerging developments such as radiomics and artificial intelligence that may further enhance its diagnostic and prognostic value.

## Clinical application of CCT

While CMR remains the preferred imaging modality for cardiomyopathy due to its superior tissue characterization capabilities [1], CCT is increasingly recognized as a valuable alternative. It offers a multiparametric approach including: (a) evaluation of coronary anatomy; (b) functional assessment with quantification of ventricular volumes and ejection fraction; (c) detection of focal myocardial fibrosis using the late iodine enhancement (LIE) technique; (d) assessment of diffuse myocardial fibrosis through extracellular volume (ECV) quantification; and (e) support for treatment planning and post-treatment evaluation [19].

### Coronary anatomy

**Fig. 1:**
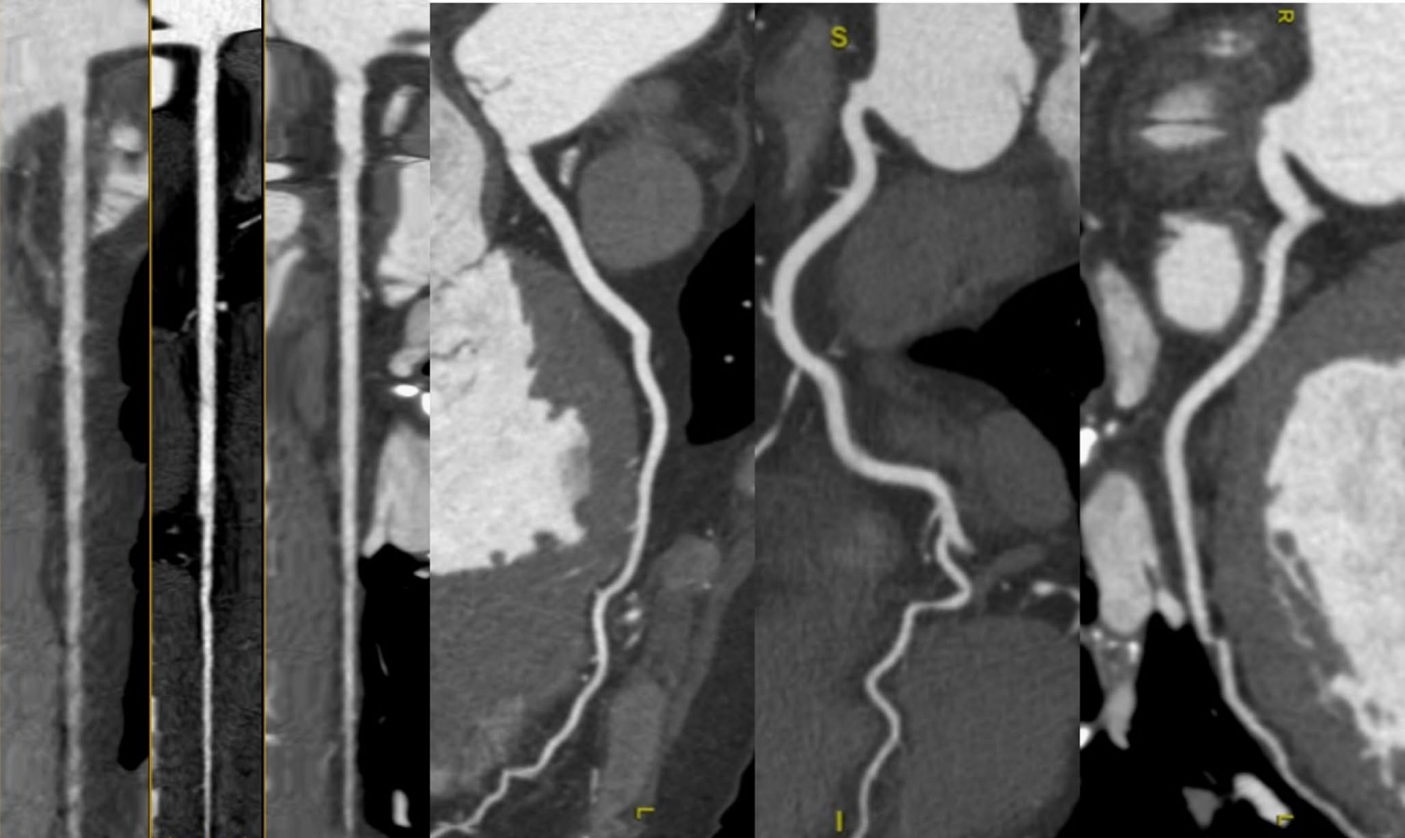
Multiplanar linear and curved reconstructions of the coronary arteries in a patient with hypertrophic cardiomyopathy

Cardiomyopathies are defined as myocardial disorders in which the heart muscle is structurally and functionally abnormal in the absence of secondary causes, including coronary artery disease [1]. Therefore, the initial step in the diagnostic work-up of patients with suspected cardiomyopathy or new-onset heart failure to exclude an ischemic etiology [1]. In this context, CCT has emerged as a highly effective, non-invasive imaging modality for the evaluation of coronary anatomy (Fig. 1). One of its most notable strengths is its high specificity (95–98%) and excellent negative predictive value (95–100%), making it particularly suitable for ruling out coronary artery disease in patients with low to intermediate pre-test probability [20–22]. Current European Society of Cardiology (ESC) guidelines for both acute and chronic heart failure recommend the use of CCT in patients with low to intermediate likelihood of coronary artery disease or in those with inconclusive or equivocal non-invasive stress tests (Class IIa recommendation) [23]. Importantly, CCT has been assigned a high appropriateness rating not only in patients with ischemic symptoms but also in those with heart failure of uncertain etiology, regardless of symptom presentation [24]. Beyond its diagnostic utility, CCT offers precise anatomical visualization of coronary artery origin, course, and potential anomalies, which may also contribute to the clinical presentation in selected cases [19].

### Function

Thanks to its isotropic spatial resolution, high temporal resolution, and excellent contrast between the ventricular lumen and myocardium, CCT allows precise assessment of cardiac chamber dimensions, global and regional function, wall motion, and left ventricular mass [19, 25–29].

**Fig. 2:**
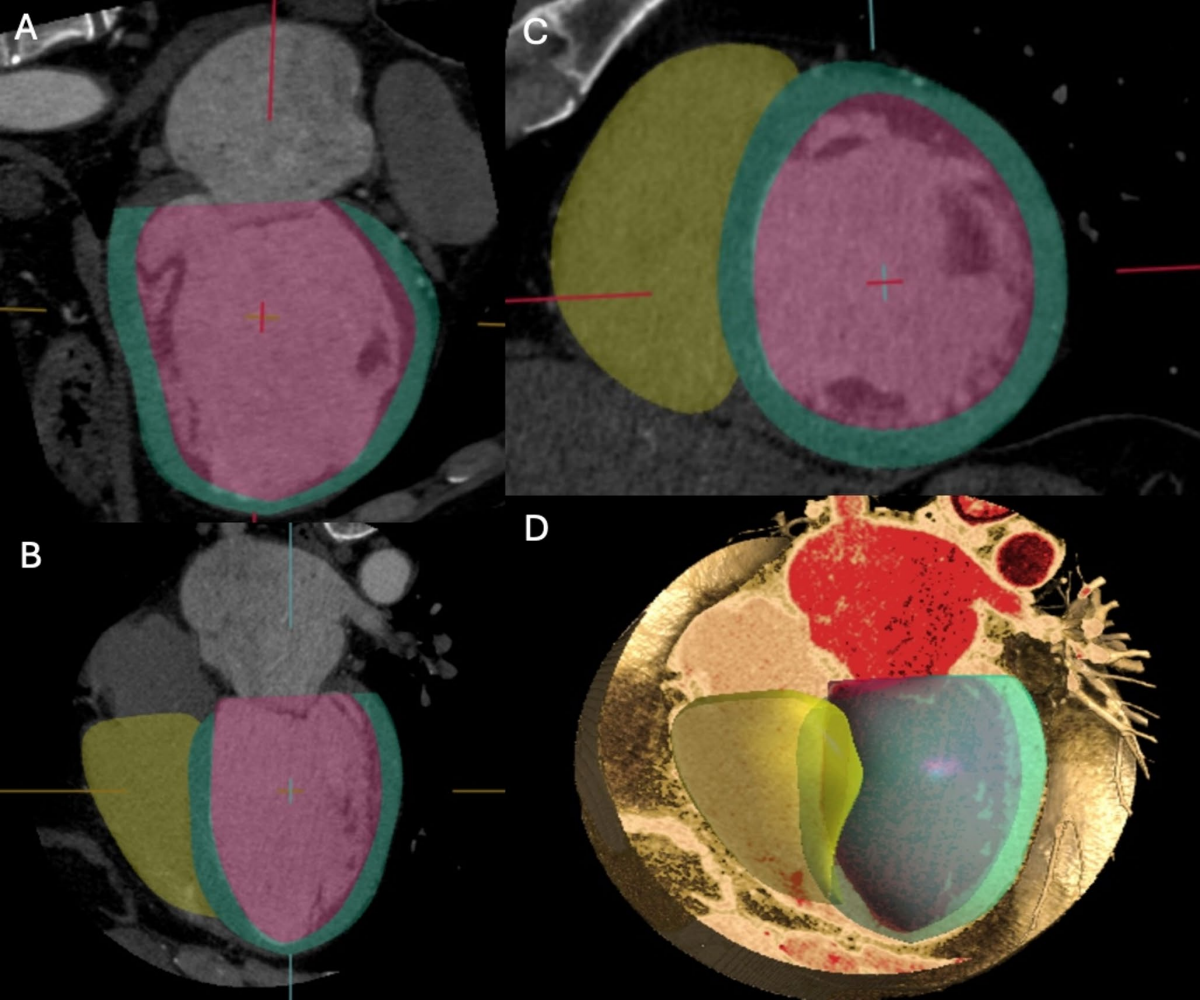
Practical example of left and right ventricular volume and function assessment using computed tomography. Endocardial and epicardial borders of the left ventricle are delineated with red and green contours, respectively, in both long-axis (Panels** A** and **B**) and short-axis views (Panel **C**). Yellow lines in Panel C indicate the endocardial borders of the right ventricle. Panel **D** shows a volumetric rendering of a four-chamber view with an endoluminal perspective. Images were post-processed using Circle CVI software

Figure 2. Numerous studies have validated the accuracy of CCT-derived functional parameters, demonstrating excellent agreement with CMR, the current reference standard. In a systematic review and meta-analysis by Kaniewska et al., which included 53 studies and 1,814 patients, the diagnostic accuracy of CCT for assessing global and regional LV function was compared with that of CMR [27]. The mean difference between CCT and CMR was minimal across key parameters: −0.56% for ejection fraction (EF), 2.62 mL for end-diastolic volume (EDV), 1.61 mL for end-systolic volume (ESV), 3.21 mL for stroke volume (SV), and 0.13 g for LV mass, with narrow limits of agreement [27]. Moreover, CCT demonstrated high diagnostic performance in detecting wall motion abnormalities, with 90% sensitivity and 97% specificity on a per-segment basis [27, 30, 31]. Beyond the LV, CCT also allows for reliable assessment of right ventricular (RV) volumes and function, again showing strong correlation with CMR [30, 31]. Emerging applications include the use of CCT for myocardial strain analysis—offering evaluation of longitudinal, radial, and circumferential deformation patterns. This may provide additional insight into subclinical myocardial dysfunction, even in patients with preserved EF, with diagnostic performance comparable to both echocardiography and CMR [12, 32–35].

### Valvular assessment

In addition to coronary anatomy and myocardial function assessment, CCT provides high-resolution visualization of cardiac valves, enabling detailed evaluation of valve morphology, leaflet thickening, annular dimensions, leaflet length, calcification, and spatial relationships with adjacent structures. It also allows dynamic assessment of valvular motion throughout the cardiac cycle, making it an essential tool for the heart valve team to complement echocardiography and to guide decision-making regarding suitability and mode of intervention [36, 37]. Retrospective CCT acquisitions can provide valuable insights into the impact of cardiomyopathy on valve function, emphasizing the bidirectional interplay between cardiomyopathy and valvular disease, which often creates a vicious cycle as seen in dilated and hypertrophic cardiomyopathy [38].

### Tissue characterization

**Fig. 3:**
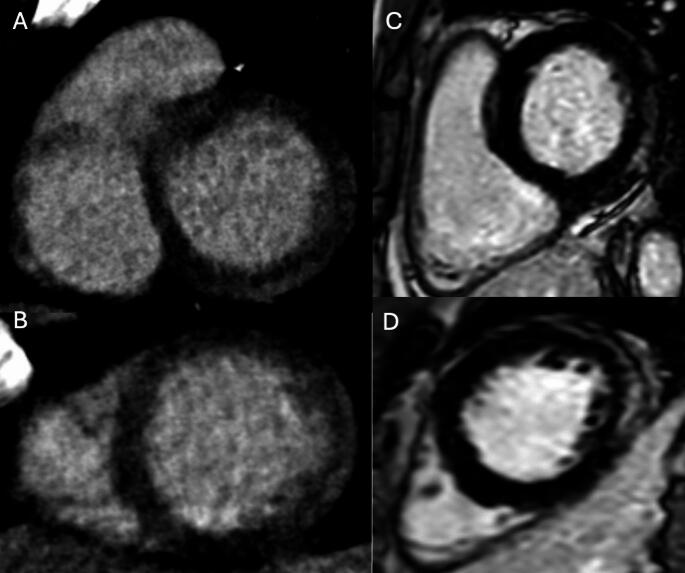
Comparison of late iodine enhancement (LIE) on cardiac CT and late gadolinium enhancement (LGE) on cardiac magnetic resonance (CMR) in a patient with acute myocarditis. Basal and mid-ventricular short-axis CT images (Panels **A **and **B**) show subepicardial late iodine enhancement in the anterolateral and inferolateral walls. Corresponding CMR images in the same short-axis views (**C** and **D**) confirm the presence and distribution of LGE in the same myocardial segments

**Fig. 4:**
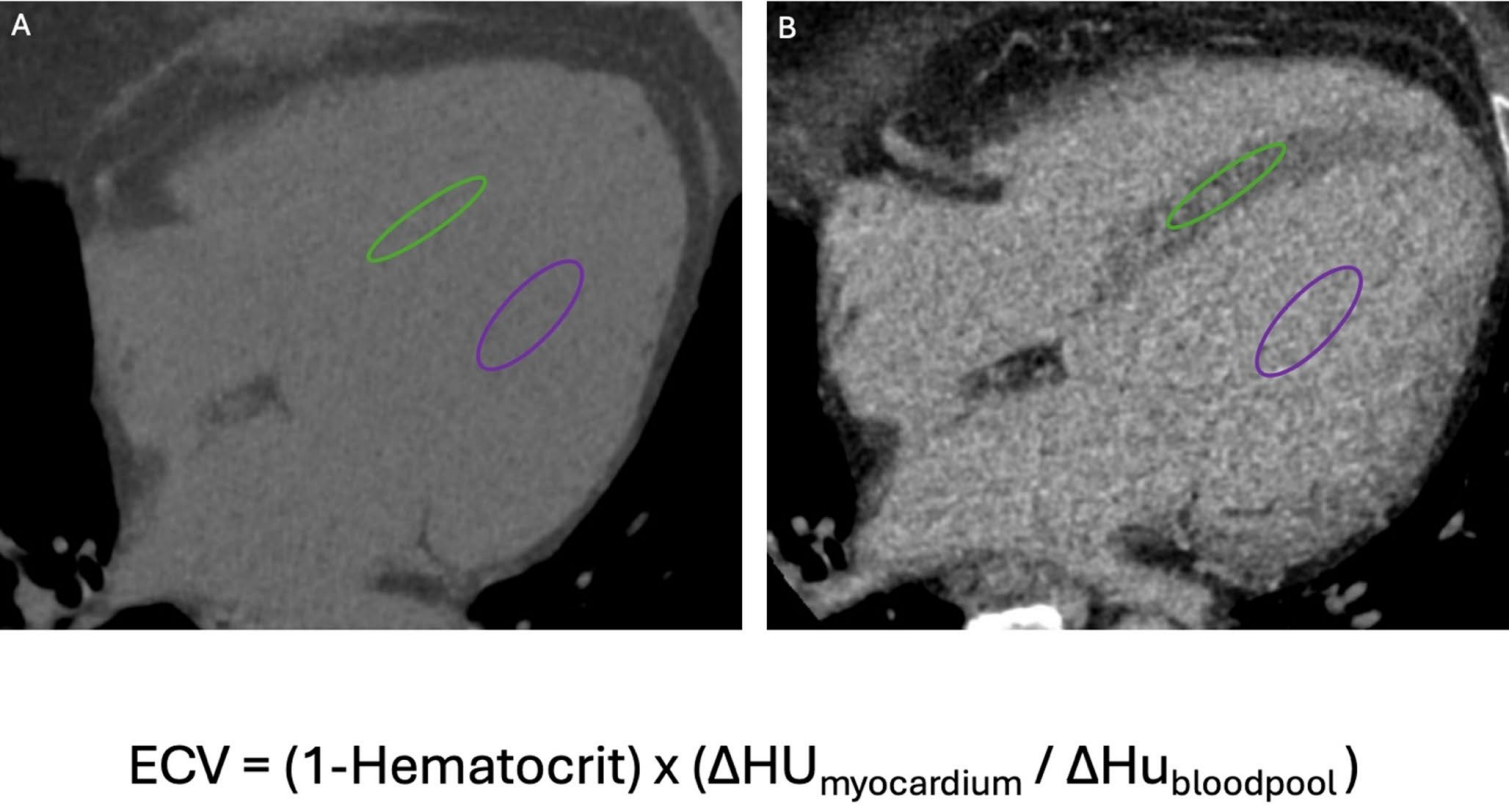
Extracellular volume (ECV) quantification using computed tomography. The calculation of ECV is based on the ratio between the concentration of contrast agent in the myocardium and in the plasma component of the blood pool. Therefore, measuring the patient’s hematocrit is essential to convert whole blood concentrations into plasma concentrations. Hounsfield unit (HU) measurements are obtained by placing regions of interest (ROIs) in the left ventricular cavity (for blood pool values, purple ellipse in Panels **A **and **B**) and in the myocardium (green ellipse in Panels **A** and **B**), carefully avoiding papillary muscles and trabeculae. This allows for the assessment of extracellular contrast agent uptake in both pre-contrast (Panel **A**) and post-contrast (Panel **B**) scans. The differences in HU (∆HU) between pre- and post-contrast images are then used to evaluate the distribution of contrast agent, according to the formula shown in the lower panel

**Fig. 5:**
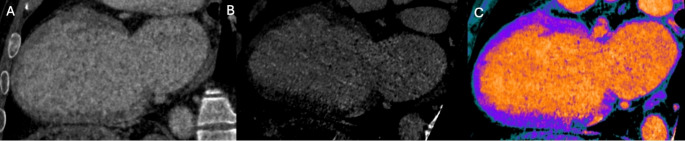
Dual-energy CT algorithm for myocardial tissue characterization. Panel **A** shows a faint area of late iodine enhancement (LIE) in the basal inferior segment with a non-ischemic distribution pattern on CT images acquired at 80 kV. Dual-energy reconstruction at low virtual monochromatic energy (55 keV) enhances iodine conspicuity, allowing clearer visualization of LIE (Panel **B**). The corresponding two-chamber iodine map (Panel **C**) further confirms the location and extent of iodine uptake

Recent technological advancements have positioned CCT as a valuable imaging modality for myocardial tissue characterization through LIE and ECV quantification [39–41]. LIE exploits the delayed washout of iodinated contrast in regions of myocardial fibrosis or scar, producing hyperdense areas analogous to late gadolinium enhancement on CMR [42], since both gadolinium and iodinated contrast media share similar pharmacokinetic properties [43]. Figure 3. This technique enables the differentiation of various patterns of myocardial fibrosis in cardiomyopathy. CT-derived ECV, on the other hand, quantifies myocardial extracellular expansion by assessing the relative iodine concentration in the myocardium and blood pool, corrected for the patient’s hematocrit [41]. Figure 4. This quantitative approach allows for the detection of diffuse interstitial fibrosis, even in the absence of discrete focal scar. Innovations in dual-energy CT (DECT) have further enhanced the clinical utility of CCT in this context, allowing for the identification of different materials based on their distinct x-ray absorption at high and low energy levels, using virtual native and iodine density imaging [39–42]. Figure 5. Furthermore, unlike traditional single-energy techniques, DECT allows spectral ECV measurement to be obtained from a single contrast-enhanced acquisition, eliminating the need for a separate pre-contrast scan [39].

**Fig. 6:**
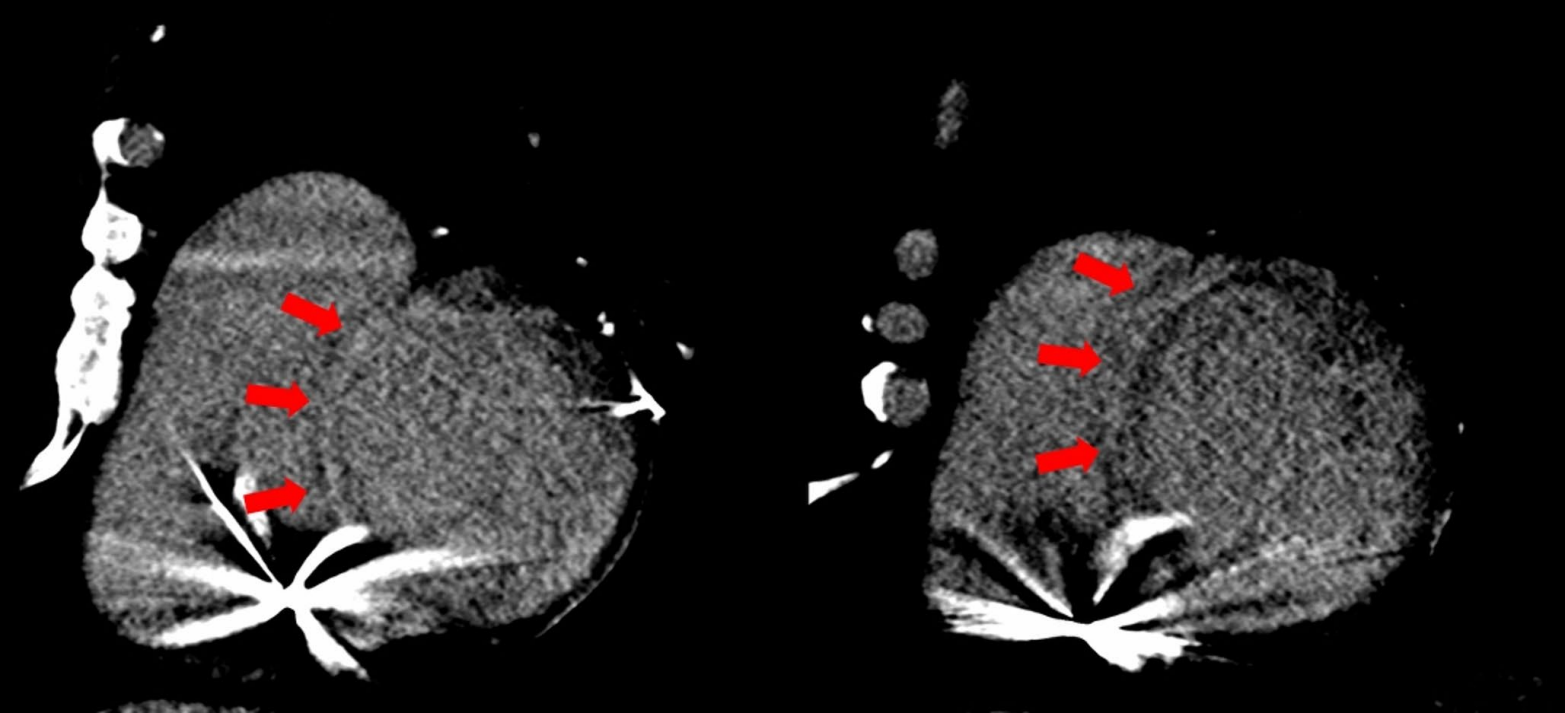
Tissue Characterization with late iodine enhancement (LIE) imaging in a Patient with Ischemic Dilated Cardiomyopathy and a CMR-Incompatible Device. Short-axis cardiac CT images acquired during the delayed phase demonstrate LIE with a transmural distribution, consistent with ischemic scar involving the septal walls

Additionally, CCT tissue characterization capabilities offer a valuable alternative in patients with CMR-incompatible devices, allowing noninvasive assessment of myocardial fibrosis. Figure 6.

### CCT limitations

Despite its growing role in the diagnostic evaluation of cardiomyopathies, CCT presents several limitations. One of the primary drawbacks is the exposure to ionizing radiation, which may be of particular concern in younger patients or in scenarios requiring serial imaging. In addition, CCT requires iodinated contrast, making it unsuitable for patients with contraindications such as severe contrast allergies or advanced renal impairment. Furthermore, certain advanced imaging techniques—such as Virtual Monoenergetic Imaging and Iodine Perfusion Maps [44]—require dual-energy CT technology, which is not yet widely available in all clinical settings, limiting the broader applicability of more refined diagnostic protocols.

## CCT protocol in suspected cardiomyopathy

The CCT protocol should be tailored to the clinical question, balancing diagnostic yield against radiation. A comprehensive protocol typically includes three sequential phases:

1. Non-contrast scan.
2. Contrast-enhanced CCT angiography.
3. LIE imaging performed 5–10 min post-contrast.

Electrocardiogram (ECG) gating is essential across all acquisitions, with prospective gating preferred for non-contrast and LIE scans to reduce radiation exposure. Conversely, retrospective ECG gating—despite higher radiation burden—is recommended during CCT angiography when functional assessment is required, because it enables accurate quantification of ventricular volumes and ejection fractions [19]. When a retrospective acquisition is chosen, tube-current or tube-voltage modulation should be applied to the portions of the cardiac cycle that are not required for coronary artery analysis, thereby limiting unnecessary radiation dose. Contrast injection protocols should follow established guidelines, such as those from the Society of Cardiovascular CT [45]. For coronary arteries and left ventricular evaluation, a biphasic injection protocol is used, typically consisting of 50–120 mL of contrast at 5–7 mL/s, followed by a 40–50 mL saline flush, achieving optimal opacification of the left heart and coronary arteries while minimizing contrast in the right heart and superior vena cava. For right ventricular assessment, a triphasic protocol is recommended: an initial contrast bolus at 5–7 mL/s is followed by either a slower contrast phase (2 mL/s) or a 50:50 contrast-saline mixture at 5–7 mL/s, and finally a smaller saline bolus. This approach ensures homogeneous opacification of all cardiac chambers while minimizing streak artifacts in the superior vena cava [45].

Because LIE imaging relies on the differential wash-out of iodine between normal and abnormal myocardial tissue—similarly to other parenchymal organs—a higher iodine dose than that used for the angiographic phase is required. Current evidence suggests that an iodine dose in the range of 0.55–0.60 g I/kg provides an optimal balance between contrast enhancement and overall image quality [9, 18].

## Current classification of cardiomyopathy

Cardiomyopathies are a heterogeneous group of myocardial disorders, defined as conditions in which the heart muscle is structurally and functionally abnormal, in the absence of coronary artery disease, hypertension, valvular disease, or congenital heart anomalies sufficient to explain the observed myocardial dysfunction [1]. The current classification, endorsed by the ESC, categorizes these conditions into five major phenotypes: hypertrophic cardiomyopathy (HCM), dilated cardiomyopathy (DCM), restrictive cardiomyopathy (RCM), arrhythmogenic cardiomyopathy (ACM), and the more recently described non-dilated left ventricular cardiomyopathy (NDLVC) [1]. A major innovation in the latest ESC guidelines is the emphasis on myocardial tissue characterization through non-invasive imaging, underscoring the central role of a multimodality imaging approach in accurate phenotyping, risk stratification, and clinical decision-making. While CMR remains the gold standard for non-invasive tissue characterization—earning a Class I, Level B recommendation in patients with suspected cardiomyopathy, CCT is increasingly recognized for its complementary role [1]. In clinical practice, CCT is primarily employed to exclude coronary artery disease) in patients with suspected cardiomyopathy, where CAD may represent a differential diagnosis or a coexisting condition. Beyond coronary assessment, CCT also contributes valuable ancillary information, including the detection of congenital coronary or vascular anomalies, concomitant pulmonary disease, pericardial pathology, and chest wall deformities [1]. In the following section, we will explore the emerging applications and supporting evidence for the use of CCT, highlighting its evolving role within the broader imaging algorithm for cardiomyopathy evaluation. Table 1 summarize previous studies about the role of CCT in cardiomyopathy.

**Table 1:** Previous studies about the role of CCT in cardiomyopathy

Authors	Years	Number of patients enrolled	Cardiomyopathy	Results
Zhao et al.	2013	47	HCM	Strong correlation in wall thickness (*r* = 0.91) and good agreement in fibrosis detection between LIE and LGE-CMR
Langer et al.	2014	24	HCM	Strong correlation between LIE and LGE-CMR for fibrosis quantification (*r* = 0.93); 100% sensitivity at patient level using a 17-segment model
Baggiano et al.	2023	39	DCM	Strong correlation with CMR-ECV (*r* = 0.819); Bland-Altman bias: 2.1%; excellent intra- and inter-observer reproducibility (ICC 0.986 and 0.966)
Yashima et al.	2023	70	DCM	Higher ECV in patients with MACE (37.16% vs. 32.59%); ECV ≥ 32.26% predicted MACE; ECV was the only independent predictor (*P* = 0.0354)
Nakajima et al.	2013	77	ARVC	CT-based score for fatty infiltration, bulging, RV dilatation: sensitivity 77.8%, specificity 96.0%, accuracy 89.6%
Venlet et al.	2020	41	ARVC	RV subepicardial tissue heterogeneity (AUC: 0.97); threshold 25 HU/mm identified LP + regions (AUC: 0.80); distinguished ARVC from controls and exercise-induced remodeling
Aikawa et al.	2017	24	NDLVC (Sarcoidosis)	Strong correlation with LGE-CMR (*r* = 0.96 per patient, *r* = 0.83 per segment); unaffected by devices; sensitivity 94%, specificity 33%
Palmisano et al.	2022	84	Acute chest pain	LIE imaging increased diagnostic yield from 50% to 90%; facilitated diagnosis of myocarditis, Takotsubo, amyloidosis, MINOCA, DCM
Andreini et al.	2023	94	Left ventricle dysfunction	CCT showed excellent concordance with CMR in identifying the etiology of left ventricular dysfunction (94.7%, 89 out of 94 patients). The diagnostic yield of CCT for detecting LIE was also high (96.7%, 1,544 out of 1,598 myocardial segments) and comparable to that of CMR (97.4%; *P* = 0.345)
Kidoh et al.	2023	552	Amyloidosis	Excellent diagnostic performance (AUC = 0.97); sensitivity 90%, specificity 92% in identifying cardiac amyloidosis

### Hypertrophic cardiomyopathy

HCM is characterized by increased left ventricular wall thickness, which may also involve the right ventricle. Importantly, the degree of hypertrophy observed cannot be solely explained by abnormal loading conditions such as hypertension or valvular disease [1]. The utility of a comprehensive CCT protocol in the assessment of HCM has been investigated by Zhao et al. Specifically, their study focused on the accuracy of CCT in measuring myocardial wall thickness. Compared to CMR, CCT demonstrated excellent correlation in wall thickness quantification (*r* = 0.91) [46]. Beyond morphologic assessment, the authors also evaluated LIE for the detection of myocardial fibrosis. When compared with late gadolinium enhancement on CMR, LIE showed a high degree of agreement in identifying fibrotic areas, particularly in characteristic locations such as the right ventricular insertion points, where patchy mid-wall fibrosis is commonly observed in HCM [46].

These findings are further supported by a prospective single-center validation study assessing the feasibility of LIE for visualizing intramyocardial fibrosis in HCM patients. Using a 17-segment model, the study demonstrated a strong correlation between LIE and LGE-CMR in quantifying fibrotic burden (*r* = 0.93), with excellent patient-level sensitivity (100%) [47].

### Dilated cardiomyopathy

DCM is defined as left ventricular dilatation accompanied by systolic dysfunction, in the absence of coronary artery disease or hemodynamic conditions sufficient to account for the observed impairment [1].

According to ESC guidelines, CCTA is recommended in patients with suboptimal echocardiographic windows or contraindications to CMR, and to rule out congenital or acquired coronary artery disease as a potential cause of the observed myocardial abnormalities (Class IIa, Level C) [1].

The utility of CCT is increasingly being explored in the assessment of non-ischemic DCM. A recent prospective study evaluated the feasibility and accuracy of ECV quantification in 39 patients with newly diagnosed DCM (left ventricular ejection fraction < 50%) undergoing clinically indicated cardiovascular magnetic resonance (CMR). CCT enabled comprehensive myocardial segment analysis and demonstrated strong concordance with CMR-derived ECV values (*r* = 0.819, *p* < 0.001) [48]. Although CCT slightly underestimated ECV compared to CMR (31.8 ± 6.5% vs. 33.9 ± 8.0%), the inter-modality agreement was robust, with a Bland-Altman bias of only 2.1%. Furthermore, intra- and inter-observer reproducibility for CCT-derived ECV was excellent, with intraclass correlation coefficients (ICC) of 0.986 and 0.966, respectively [48].

Beyond its diagnostic capabilities, CT-derived ECV quantification also holds prognostic relevance. In this context, Yashima et al. investigated the prognostic significance of ECV assessed by CCT in a cohort of 70 patients with DCM. All patients underwent CCT with available LIE imaging and were followed for the occurrence of major adverse cardiac events (MACE), including cardiovascular death, life-threatening arrhythmias, and heart failure-related hospitalizations [49]. The study demonstrated that patients who experienced MACE had significantly higher myocardial ECV values compared to those without adverse events (mean ECV: 37.16 ± 5.91% vs. 32.59 ± 3.95%, respectively). Receiver operating characteristic (ROC) curve analysis identified an optimal ECV threshold of 32.26% for predicting MACE. Patients with ECV values ≥ 32.26% had a significantly higher cumulative incidence of adverse outcomes, as demonstrated by Kaplan–Meier survival analysis (*P* = 0.0032) [49]. Importantly, multivariate Cox proportional hazards analysis revealed that ECV was the sole independent predictor of MACE among the clinical and imaging parameters assessed (*P* = 0.0354) [49].

### Arrhythmogenic right ventricular cardiomyopathy

ARVC is characterized by right ventricular dilatation and/or dysfunction, typically associated with histopathological evidence of fibrofatty myocardial replacement and electrocardiographic abnormalities [1], as outlined in the revised Task Force criteria [50].

CCT is also gaining attention in the assessment of ARVC. Thanks to its isotropic spatial resolution, high temporal resolution, and excellent contrast between the ventricular lumen and myocardium, CCT provides detailed visualization of cardiac chamber size, wall motion, and fat infiltration. A study by Nakajima et al. evaluated 77 patients with either a confirmed or suspected diagnosis of ARVC using contrast-enhanced and non-contrast CCT. The authors proposed a novel CT-based scoring system that incorporates three hallmark features: fatty tissue infiltration, bulging morphology, and RV dilatation. The CT-derived score demonstrated excellent diagnostic performance for identifying both definite and borderline ARVC, with a sensitivity of 77.8%, specificity of 96.0%, and overall accuracy of 89.6% [51]. Recently, Venlet et al. investigated the utility of CCT-derived RV tissue heterogeneity in distinguishing ARVC from exercise-induced arrhythmogenic remodeling and healthy controls. By integrating electroanatomic voltage mapping with CT imaging, they found that RV subepicardial tissue heterogeneity was significantly greater at sites with late potentials (LP+), which are indicative of arrhythmogenic substrate. A CT heterogeneity threshold of 25 HU/mm demonstrated good diagnostic performance for identifying LP + regions (AUC: 0.80). Furthermore, global CT-derived tissue heterogeneity effectively differentiated ARVC from controls (AUC: 0.97; sensitivity: 100%; specificity: 82%) and from exercise-induced arrhythmogenic remodeling (AUC: 0.78; sensitivity: 65%; specificity: 89%) [52].

### Non-Dilated left ventricular cardiomyopathy

**Fig. 7:**
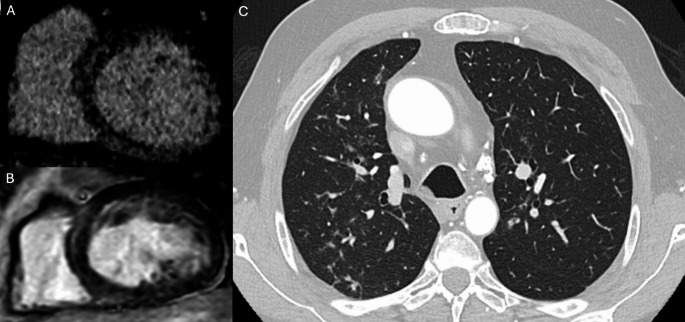
Illustrative example of cardiac sarcoidosis on CT and MRI. Late iodine enhancement CT image in short-axis view at the mid-ventricular level (Panel A) shows intramyocardial delayed enhancement in the interventricular septum. Corresponding short-axis late gadolinium enhancement (LGE) image on CMR (Panel B) confirms myocardial fibrosis in the same location. Axial CT reconstruction (Panel C) demonstrates partially calcified mediastinal and hilar lymphadenopathy (arrowheads), consistent with sarcoid-related adenopathy, along with perilymphatic irregular nodular thickening in an upper/mid lung distribution, suggestive of pulmonary involvement

NDLVC represents a newly proposed category within the cardiomyopathy spectrum. It is defined by the presence of regional or global left ventricular systolic dysfunction in the absence of significant chamber dilatation, accompanied by non-ischemic myocardial myocardial fibrosis [1]. This category encompasses a heterogeneous group of patients previously classified under various overlapping entities, including non-dilated forms of dilated cardiomyopathy, arrhythmogenic left ventricular cardiomyopathy, left-dominant arrhythmogenic cardiomyopathy, and arrhythmogenic dilated cardiomyopathy [1]. CCT, particularly when combined with LIE imaging, may represent a valuable tool in the diagnostic workup of myocardial diseases characterized by fibrosis. LIE enables the noninvasive detection and characterization of myocardial fibrosis and may assist in distinguishing between different etiologies based on the distribution and pattern of fibrotic involvement—ranging from acquired conditions such as post-myocarditis cardiomyopathy to infiltrative or inflammatory diseases like cardiac sarcoidosis. Aikawa et al. conducted a study to evaluate the diagnostic performance of CCT in detecting cardiac sarcoidosis, comparing it directly with CMR. The cohort included 24 patients, both with and without implantable devices. The study demonstrated excellent interobserver agreement in identifying myocardial fibrosis using LIE, unaffected by device-related artifacts [53]. Moreover, a strong correlation was observed between the extent of myocardial involvement assessed by LIE and LGE, with a per-patient correlation coefficient of 0.96 and a per-segment coefficient of 0.83 (both *p* < 0.001). In diagnosing cardiac sarcoidosis, CCT achieved a high sensitivity of 94%, although specificity remained modest at 33% [53]. Figure 7. Further reinforcing this role, a prospective study investigated a comprehensive CT protocol combining CT angiography with LIE imaging in patients presenting with acute chest pain [54]. The delayed-phase acquisition enabled myocardial tissue characterization, which proved critical in identifying underlying cardiac conditions [54]. Specifically, the distribution and pattern of LIE facilitated the diagnosis of myocarditis (52%), Takotsubo syndrome (10%), amyloidosis, myocardial infarction with non-obstructive coronary arteries (MINOCA), and dilated cardiomyopathy. The addition of LIE imaging significantly increased the overall diagnostic yield from 50% to 90% (*p* < 0.001) [54]. Similarly, a recent study assessed the feasibility and diagnostic accuracy of a comprehensive functional and anatomical evaluation using CCT compared with CMR in patients with newly diagnosed left ventricular dysfunction. Among the 94 patients included, CCT and CMR demonstrated excellent concordance (94.7%) in identifying the underlying cause of dysfunction, including 100% agreement in diagnosing ischemic cardiomyopathy. LIE was reliably detected by CCT, with a diagnostic accuracy of 94.8% and no significant difference compared to CMR [55].

### Restrictive cardiomyopathy

RCM is characterized by impaired ventricular filling due to increased myocardial stiffness, leading to restrictive physiology. It typically presents with non-dilated left and/or right ventricles, normal ventricular wall thickness, and normal or reduced diastolic and/or systolic volumes in one or both ventricles [1]. Amyloidosis is one of the leading causes of restrictive cardiomyopathy, and is a progressive infiltrative disorder characterized by the extracellular deposition of insoluble amyloid fibrils. Recent studies have highlighted the emerging role of CCT, particularly through ECV quantification, in the non-invasive diagnosis of cardiac amyloidosis [56, 57]. Kidoh et al. conducted a retrospective analysis assessing the diagnostic utility of CT-derived ECV in patients undergoing evaluation for suspected cardiomyopathy or heart failure. The study included 41 patients with confirmed cardiac amyloidosis (38 with wild-type transthyretin amyloidosis and 3 with AL amyloidosis) and 511 patients without evidence of amyloid disease [57]. The results demonstrated excellent diagnostic performance for CT-derived ECV in identifying cardiac amyloidosis, with an area under the receiver operating characteristic curve (AUC) of 0.97, sensitivity of 90%, and specificity of 92% [57]. Similarly, in a separate study involving 109 patients with aortic stenosis and suspected transthyretin-related amyloidosis, as well as 20 control subjects, CT-derived ECV also proved effective in differentiating amyloidosis from non-infiltrative myocardial thickening, achieving an AUC of 0.87 [56]. These individual findings are further supported by a recent comprehensive systematic review and meta-analysis evaluating the clinical value of CT-derived ECV across cardiovascular conditions, especially in the preoperative assessment of patients undergoing transcatheter aortic valve replacement, where differentiating amyloidosis from pressure-overload–induced hypertrophy is essential [58]. CT-ECV demonstrated excellent diagnostic accuracy for identifying cardiac amyloidosis, with a pooled sensitivity of 92.8% (95% CI: 86.7%–96.2%), specificity of 84.8% (95% CI: 68.6%–93.4%), and an AUC of 0.94 (95% CI: 0.88–1.00) [58].

## Future perspective

Radiomics has emerged as a promising tool to enhance the diagnostic potential of CCT in cardiomyopathy by extracting high-dimensional, quantitative features that are often invisible to the human eye [59–62]. Mannil et al. demonstrated that texture analysis applied to non-contrast, low-radiation-dose cardiac CT could detect myocardial fibrosis in patients with acute and chronic myocardial infarction, achieving moderate accuracy despite visually undetectable findings (AUC 0.78, sensitivity 86%, specificity 81%) [63].

Alongside radiomics, artificial intelligence (AI) is expected to play a pivotal role in transforming the clinical application of cardiac CT. Beyond its established roles in image acquisition, reconstruction, and post-processing [64–67], AI can be integrated with radiomics to efficiently manage large and complex imaging datasets [59]. Kay et al. developed a fully automated pipeline combining left ventricular segmentation, radiomic feature extraction, and machine learning to identify high-risk left ventricular hypertrophy phenotypes—defined by CMR—using only non-contrast CT data in a large population cohort. Their approach successfully leveraged underutilized information embedded in standard CT scans, potentially enabling early detection without additional imaging or radiation exposure [61].

Cavallo et al. further demonstrated the feasibility of CT-based radiomics in characterizing myocardial remodeling in patients with arterial hypertension. Their ensemble machine learning (EML) model, built on 377 extracted radiomic features, showed a significant correlation with left ventricular septum width—a surrogate marker of myocardial remodeling—supporting radiomics’ potential for phenotyping hypertensive heart disease [60].

The recent advent of photon-counting CT has introduced new opportunities in spectral imaging [14, 68–73]. This technology employs a novel generation of detectors capable of distinguishing X-ray photons based on their energy levels. Unlike conventional CT, photon-counting CT can accurately differentiate three or more materials and enables multi-energy spectral imaging without spectral overlap [14, 69, 72–75]. An additional advantage of these detectors is their ability to incorporate materials with distinct K-edge energies into spectral decomposition, paving the way for the use of alternative contrast agents beyond iodine, including gold, silver, platinum, bismuth, ytterbium, and novel nanoparticles [11, 76–79]. From a cardiomyopathy perspective, photon-counting CT holds promise to substantially improve test characteristics by enhancing tissue characterization, particularly in identifying replacement fibrosis, interstitial fibrosis, fat, and edema. Early studies suggest that improved spatial resolution, reduced image noise, and more precise quantification of myocardial attenuation may translate into better detection of subtle structural changes and more accurate phenotyping of different cardiomyopathy subtypes [13, 42, 75, 80].

Although clinical data in cardiomyopathy are still limited, the emerging literature indicates that photon-counting CT has the potential to expand diagnostic accuracy and broaden its applicability, especially in patients with contraindications to CMR.

## Conclusion

CCT has evolved beyond its traditional role in coronary artery assessment and now offers valuable contributions to the comprehensive evaluation of cardiomyopathies. In selected clinical scenarios, particularly when CMR is contraindicated, CCT serves as a powerful adjunctive tool. Its ability to provide high-resolution anatomical data, assess atrial and ventricular function and dimensions, and detect myocardial fibrosis through LIE, as well as changes in ECV, enhances its diagnostic utility within a multimodal imaging strategy.

When appropriately integrated into the diagnostic pathway, CCT can aid in the phenotyping of undifferentiated cardiomyopathy, supporting accurate diagnosis, risk stratification, and tailored clinical management. As technology advances and evidence continues to grow, CCT is expected to play an increasingly prominent role in the imaging algorithm for cardiomyopathies, complementing both echocardiography and CMR.

## Data Availability

No datasets were generated or analysed during the current study.
